# Supplementary material for: Self-harm and suicide prevention in humanitarian and fragile contexts: A systematic scoping review
Source: Glob Ment Health (Camb). 2025 Dec 4;12:e145. doi: 10.1017/gmh.2025.10108 (PMC12720389; doi:10.1017/gmh.2025.10108)
Supplement: Zemp et al. supplementary material [file S2054425125101088sup001.zip › S2054425125101088sup002.docx]

Final search terms
Self-harm and suicide prevention in humanitarian and fragile contexts: A systematic scoping review.
12 November 2024

# MEDLINE (Ebsco)

(MH "Self-Injurious Behavior+" OR TI(automutilat* OR "cutting" OR nonsuicid* OR overdos* OR parasuicid* OR poison* OR selfdestruct* OR selfharm* OR selfimmolat* OR selfinflict* OR selfinjur* OR selfmutilat* OR selfwound* OR (self N1 (destruct* OR harm* OR immolat* OR inflict* OR injur* OR mutilat* OR wound*)) OR suicid*) OR AB(automutilat* OR "cutting" OR nonsuicid* OR overdos* OR parasuicid* OR poison* OR selfdestruct* OR selfharm* OR selfimmolat* OR selfinflict* OR selfinjur* OR selfmutilat* OR selfwound* OR (self N1 (destruct* OR harm* OR immolat* OR inflict* OR injur* OR mutilat* OR wound*)) OR suicid*))

AND

(MH "Disasters+" OR MH "COVID-19+" OR MH "Warfare and Armed Conflicts+" OR TI(((armed OR civil* OR political*) N2 (conflict* OR instability OR unstable OR violen*)) OR attack* OR avalanche* OR cholera OR ((complex OR "public health") N2 emergenc*) OR COVID OR cyclone* OR disaster* OR drought* OR dysentery OR earthquake* OR ebola OR epidemic* OR explosion* OR (extreme N2 (climate OR temperature* OR weather)) OR famine* OR flood* OR fragile OR gang* OR hurricane* OR humanitarian OR "internally displaced" OR landslide* OR massacr* OR pandemic* OR postconflict OR revolution* OR shooting* OR siege* OR starv* OR storm* OR terror* OR tornado* OR tortur* OR tsunami* OR uprising* OR volcan* OR war OR wars OR warfare OR warzone* OR (waterborne N2 (disease* OR illness*)) OR wildfire*) OR AB(((armed OR civil* OR political*) N2 (conflict* OR instability OR unstable OR violen*)) OR attack* OR avalanche* OR cholera OR ((complex OR "public health") N2 emergenc*) OR COVID OR cyclone* OR disaster* OR drought* OR dysentery OR earthquake* OR ebola OR epidemic* OR explosion* OR (extreme N2 (climate OR temperature* OR weather)) OR famine* OR flood* OR fragile OR gang* OR hurricane* OR humanitarian OR "internally displaced" OR landslide* OR massacr* OR pandemic* OR postconflict OR revolution* OR shooting* OR siege* OR starv* OR storm* OR terror* OR tornado* OR tortur* OR tsunami* OR uprising* OR volcan* OR war OR wars OR warfare OR warzone* OR (waterborne N2 (disease* OR illness*)) OR wildfire*))

AND

(TI((affect* OR consequence* OR effect* OR efficacy OR impact* OR influenc* OR outcome*) N10 (allevi* OR guideline* OR intervention* OR polic* OR prevent* OR program* OR service* OR strateg*)) OR AB((affect* OR consequence* OR effect* OR efficacy OR impact* OR influenc* OR outcome*) N10 (allevi* OR guideline* OR intervention* OR polic* OR prevent* OR program* OR service* OR strateg*)))
